# Efficient Separation of Per- and Polyfluoroalkyl Substances (PFAS) by Organic Framework Membranes: Advances, Mechanisms, and Challenges

**DOI:** 10.3390/membranes16010019

**Published:** 2026-01-01

**Authors:** Jiawei Zhang, Baosheng Zhao, Hao Yang

**Affiliations:** 1 State Key Laboratory of Pollution Control and Resource Reuse, School of the Environment, Nanjing University, Nanjing 210023, China; jiawei_zhang@smail.nju.edu.cn (J.Z.);; 2 Institute for the Environment and Health, Nanjing University Suzhou Campus, Suzhou 215163, China

**Keywords:** organic framework membranes, Per- and polyfluoroalkyl substances, membrane fabrication, separation mechanisms, water treatment

## Abstract

Per- and polyfluoroalkyl substances (PFAS) represent a class of highly persistent environmental contaminants with exceptional chemical stability. Efficient removal of PFAS from water poses a significant challenge for the chemical industry and constitutes a critical requirement for sustainable environmental development. Membrane technology has demonstrated considerable potential in water treatment due to its low energy consumption and environmentally friendly characteristics. This review comprehensively summarizes recent advances in emerging metal–organic framework (MOF)-, covalent organic framework (COF)-, and hydrogen-bonded organic framework (HOF)-based membranes for highly efficient separation and catalytic degradation of PFAS. We provide a detailed analysis of design strategies for various organic framework membranes (OFMs) and their synergistic separation mechanisms, including size exclusion, electrostatic interactions, adsorption, as well as catalytic degradation based on advanced oxidation processes. Furthermore, we systematically evaluate the performance and applicability of these membranes in practical aquatic environments. Finally, this review outlines future directions toward developing integrated “separation-degradation” membrane processes for practical applications by discussing current challenges concerning material stability, manufacturing costs, and long-term operational efficiency. This review aims to provide theoretical guidance and technical insights for developing next-generation high-performance membranes for PFAS removal.

## 1. Introduction

Per- and polyfluoroalkyl substances (PFAS) are a class of synthetic chemicals with exceptional chemical stability; the strong carbon–fluorine bonds in their molecules render them almost non-degradable in nature, earning them the moniker “forever chemicals” [1]. Global PFAS production is estimated at ~320,000 t·yr^−1^, and the compounds are ubiquitous in plastics, textiles, coatings, electronics and numerous other industrial sectors [2]. Yet this persistence allows PFAS to accumulate indefinitely in aquatic systems, bio-magnify through food webs and pose long-term risks to ecosystems and human health [1,3,4,5].

Perfluorooctane sulfonate (PFOS) and perfluorooctanoic acid (PFOA), the archetypal long-chain PFAS, were listed under the Stockholm Convention in 2009 and 2019, respectively, and in 2023 were incorporated into China’s “List of New Pollutants under Priority Control”. As long-chain PFAS face stringent restrictions, short-chain analogues such as perfluorobutanoic acid (PFBA) and perfluorobutane sulfonic acid (PFBS) are being widely adopted on the assumption of lower bioaccumulation potential [6]. However, emerging evidence indicates that short-chain PFAS can still disrupt thyroid hormones, alter cholesterol homeostasis and elicit developmental toxicity [7], enable them to disperse over long distances in aquatic environments, thereby increasing the difficulty of pollution control [8].

Adsorption and ion-exchange technologies have been extensively employed over the past decade for removing PFAS from wastewater and groundwater [9,10]. Although these approaches show promise, they are generally effective only for PFAS within a limited chain-length range [11]. For example, when a cationic quaternized nanocellulose adsorbent is used, the maximum uptake for PFOS reaches 559 mg·g^−1^, whereas that for PFBA is merely 121 mg·g^−1^ [12]. Similarly, ion-exchange systems can achieve 92–97% removal of PFOS, but only ~14% for PFBA, and their performance is readily compromised by competing ions. Membrane technology employs a membrane as the separation medium and uses a chemical-potential gradient as the driving force, offering low energy consumption, high throughput, and high selectivity [13,14,15,16,17]. In recent years, it has demonstrated distinct advantages for PFAS removal from water [18,19]. The presence of tunable nanochannels formed by nano- and micro-voids in the membrane is crucial for the effective separation of specific components from a homogeneous mixture, a process which would be prevented or slowed by a dense, cavity-free film [20,21]. Through advanced fabrication strategies, such as controlling the dispersed phase of two-dimensional nanosheets (e.g., MXene) during interfacial polymerization, the structure, cross-linking degree, and nano-voids of the polyamide layer can be precisely tailored to optimize membrane permselectivity [22]. However, achieving higher precision in sieving and mass transfer requires membrane materials with more regular and precise pore channels.

In advancing membrane technologies for PFAS separation, organic framework membranes (OFMs) constructed from metal–organic frameworks (MOFs), covalent organic frameworks (COFs), and hydrogen-bonded organic frameworks (HOFs) have drawn growing research interest. Constructed through coordination bonds, covalent bonds, or hydrogen bonds, these porous frameworks feature well-defined structures, permanent porosity, tunable pore sizes, and customizable channels [23,24,25,26,27,28]. These properties enable flexible multiscale design, positioning OFMs as promising membranes combining high water flux with efficient PFAS rejection, thereby providing a reliable strategy against PFAS contamination.

Unlike previous reviews that have treated these frameworks in isolation or merely as nanofillers [29,30,31], this work provides a unified and systematic analysis of their shared design principles, fabrication strategies, and synergistic separation mechanisms. Specifically, the review systematically organizes the relevant fabrication methods (ranging from composite membranes to fully crystalline membranes) and delves into the synergistic interactions among key separation mechanisms, including size sieving, electrostatic interactions, and specific host–guest binding, thereby establishing a clear distinction from prior literature.

Finally, this review discusses the current critical challenges and future research directions, offering mechanism-oriented and forward-looking guidance to overcome existing obstacles and advance the development of high-performance OFMs technologies.

## 2. Fabrication Strategies and Structural Design of OFMs for PFAS Removal

Depending on the fabrication strategy, OFMs can be primarily categorized into mixed matrix membranes (MMMs), thin-film nanocomposite (TFN) membranes, and continuous OFMs. This section will elaborate on these mainstream membrane construction approaches. The limitations concerning structural defects and stability of OFMs under PFAS treatment conditions will also be discussed.

### 2.1. MMMs

As a mainstream strategy for OFMs, MMMs are fabricated by dispersing pre-synthesized framework fillers into a polymeric matrix via phase inversion [32]. This approach combines the mechanical robustness of the polymer with the molecular sieving capability of the fillers, yet it often encounters challenges such as interfacial defects, filler aggregation at high loadings, and compatibility issues [33].

**Figure 1:**
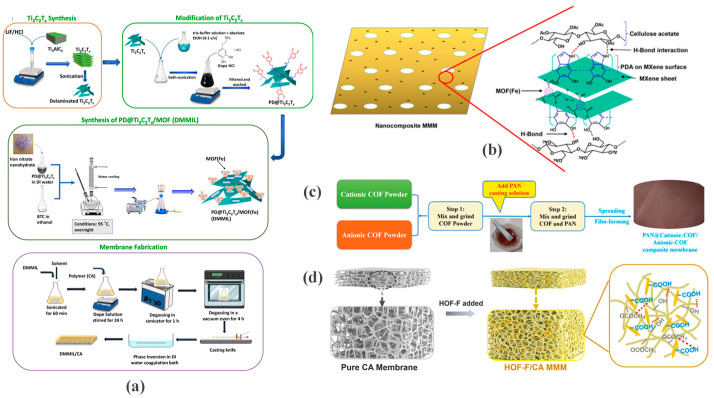
(**a**) Synthesis of nanocomposite material (DMMIL), and membrane fabrication procedure. Reproduced from [35] with Open Access; (**b**) Possible interactions between CA and PDA@Ti_3_C_2_T_x_/MOF(Fe). Reproduced from [35] with Open Access; (**c**) Preparation scheme of PAN@iCOFs composite membrane. Reprinted with copyright permission from Ref. [36] Copyright 2022 Wiley-VCH GmbH; (**d**) Process diagram for the formation of HOF-F/CA MMMs. Reprinted with copyright permission from Ref. [37] Copyright 2025 Elsevier B.V.

In MOFs-based MMMs, Zhang et al. [34] addressed the inherent limitations of conventional hydrophobic polytetrafluoroethylene (PTFE) membranes in direct contact membrane distillation (DCMD), including low PFAS/ammonia separation efficiency and membrane wetting, by coating them with a hydrophilic polyvinyl alcohol (PVA) layer containing incorporated aluminum fumarate (AlFu) MOFs. To further achieve functional synergy between different materials, Minhas et al. [35] fabricated DMMIL/CA MMMs by coating polydopamine (PDA) onto MXene nanosheets, followed by the in situ growth of MOF (MIL-100(Fe)) to form a hybrid nanofiller, which was then incorporated into a cellulose acetate matrix (Figure 1a,b). This design combined the hydrophilicity of MXene, porosity of the MOF, and adhesion of PDA to improve membrane performance.

To endow the membrane with specific charge characteristics, Zhang et al. [36] physically mixed a positively charged cationic COF (EB-COF) powder with a negatively charged anionic COF (TpPa-SO_3_Na) powder. This dual-charge filler was then dispersed in a PAN matrix (Figure 1c). This design enables the membrane surface to simultaneously possess both positive and negative charge regions, forming a zwitterion-like chemical microenvironment.

Lin et al. [37] prepared HOF-F/CA MMMs via a phase inversion method, using one-dimensional needle-shaped HOF-F as the filler and cellulose acetate (CA) as the polymer matrix. HOF-F was synthesized through low-temperature ethanol-induced self-assembly and features rhombic channels (approximately 24.92 Å × 21.38 Å) and abundant surface -COOH and -F groups (Figure 1d). Importantly, the synthesis of HOF-F demonstrated that precise control over crystal morphology through optimized solvent systems and reaction temperature is crucial for achieving uniform dispersion and reducing interfacial defects. Due to the purely organic structure of HOFs, they exhibit good compatibility with polymer matrices like CA.

### 2.2. TFN Membranes

Modulating the structure and chemical properties of the polyamide (PA) selective layer is a key strategy to enhance membrane separation performance. Introducing organic framework during interfacial polymerization helps control the microstructural construction of the PA layer.

#### 2.2.1. Integration of Organic Framework into PA Layer

**Figure 2:**
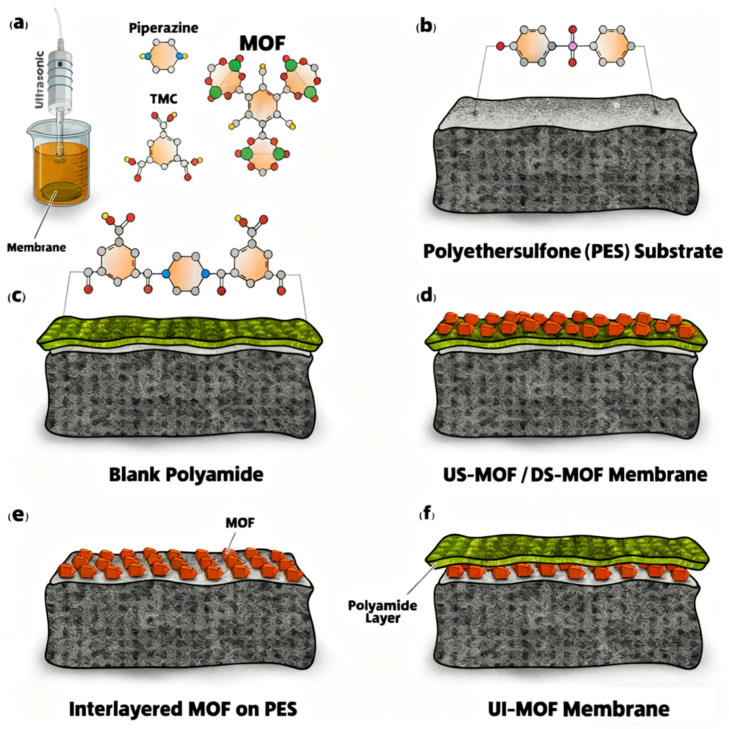
Schematic diagrams of the membrane designs. Reproduced from [39] with Open Access: (**a**) The in situ growth of MOFs on the surface, the molecular structures of key monomers (piperazine (PIP), TMC), and the synthesized MOFs; (**b**) Chemical structure of polyethersulfone (PES) support; (**c**) The fabricated blank membrane (PA/PES); (**d**) Surface-grafted MOF membranes (US-MOF, DS-MOF); (**e**) Ag-MOFs interlayer before interfacial polymerization (IP); (**f**) Interlayered MOF membrane (UI-MOF).

Bi et al. [38] fabricated a ZIF-L/PEI-TMC composite membrane by dispersing ZIF-L nanoparticles in an aqueous polyethylenimine (PEI) solution prior to reacting with trimesoyl chloride (TMC). However, when the ZIF-L loading in the ZIF-L/PEI membrane was increased to 10 wt%, it led to MOF aggregation and surface defects. This resulted in a significant decrease in PFAS rejection, despite the increased water flux. Differing from the direct embedding of nanofillers in the PA layer, Pilevar et al. [39] investigated an approach to integrate Ag-MOFs as standalone functional layers. They systematically evaluated three techniques (Figure 2): constructing an ultrasonically-assisted interlayer under the PA (UI-MOF), and grafting Ag-MOFs onto the PA surface via ultrasonication (US-MOF) or dip-coating (DS-MOF). Each technique confers unique surface chemical properties to the modified membranes, thereby influencing their surface hydrophilicity, which subsequently enhances surface wettability, water permeability, and antifouling resistance.

#### 2.2.2. Capillary-Assisted Interfacial Polymerization

**Figure 3:**
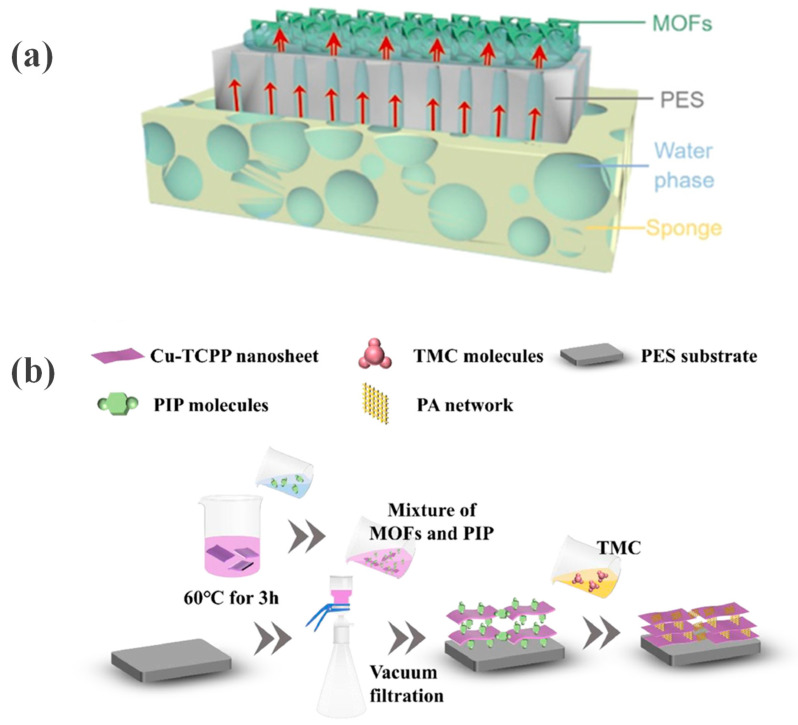
(**a**) Capillary rise of the aqueous-phase solution within the nanochannels of the PES membrane (indicated by single-headed arrows) and the MOF layer (double-headed arrows). Reprinted with copyright permission from Ref. [40] Copyright 2022 American Chemical Society; (**b**) PA-modified MOF membrane fabrication process. Reprinted with copyright permission from Ref. [41] Copyright 2024 Elsevier Ltd.

Zhao et al. [40] developed a capillary-assisted interfacial polymerization (CAIP) method, in which the PA layer is formed by transporting monomers via capillary action through a support pre-deposited with MOFs (Figure 3a). While this approach effectively tailors the membrane structure to enhance performance, its efficacy is highly sensitive to MOF loading and sponge saturation level. Inappropriate conditions can lead to MOF aggregation or incomplete PA cross-linking, thereby compromising separation efficiency and stability.

#### 2.2.3. Defect Repair Strategy for Framework Materials

Zhang et al. [41] selectively formed PA within the defects between MOF nanosheets via in situ IP (Figure 3b). The resulting MOF-PA membrane achieved efficient removal of various PFAS under high water permeance, along with significantly improved operational stability and antifouling properties. However, the adsorption of PFAS by the membrane is prone to saturation during long-term operation, which may affect its long-term treatment capacity.

### 2.3. Functionalized Support Layer

**Figure 4:**
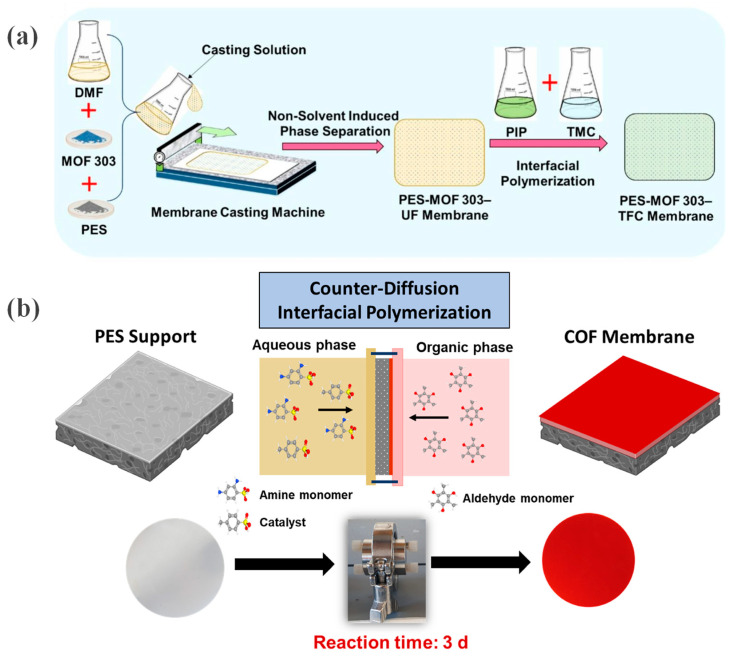
(**a**) Fabrication of PES-MOF 303-UF and PES-MOF 303-TFC membranes via IP. Reprinted with copyright permission from Ref. [42] Copyright 2025 Elsevier B.V.; (**b**) COF membrane synthesis by counter-diffusion IP at room temperature. Reprinted with copyright permission from Ref. [43] Copyright 2024 Elsevier B.V.

The functionalized support layer strategy enhances membrane performance by optimizing the porous support. Koli et al. [42] blended MOF-303 nanoparticles with polyethersulfone (PES) and *N*,*N*-dimethylformamide (DMF) to fabricate a PES-MOF-303 composite ultrafiltration support membrane via phase inversion (Figure 4a), followed by interfacial polymerization to form the polyamide separation layer.

### 2.4. Continuous OFMs

Compared to MMMs, continuous COF membranes can more effectively leverage the unique intrinsic properties of COFs. Nguyen et al. [43] employed a counter-diffusion interfacial polymerization strategy to directly grow a continuous anionic TpPa-SO_3_H COF thin film on a polymeric support (Figure 4b). In this process, the support membrane separated organic and aqueous phases containing the aldehyde and amine monomers, respectively. The monomers diffused across the support and met at its surface, where they reacted and gradually formed a complete, crystalline COF selective layer. By varying the monomer concentrations, COF layers with tunable structural characteristics were achieved.

In OFMs, crystal orientation is a key structural parameter for regulating separation performance. Studies have shown that synthesis strategies such as oriented epitaxial growth and vapor-assisted conversion can successfully produce MOF membranes with specific orientations, thereby significantly reducing grain boundary defects and optimizing molecular transport pathways [44,45]. Although research on the directional removal of PFAS remains limited, these findings clearly demonstrate that crystal orientation is tunable during membrane fabrication. Therefore, precise control of MOF crystal orientation holds the potential to simultaneously enhance the size-exclusion precision and the utilization efficiency of adsorption sites, providing a new design strategy and material basis for next-generation PFAS separation membranes that combine high rejection with high flux.

## 3. Separation Mechanisms of OFMs for PFAS Removal

### 3.1. Separation via Physical and Chemical Mechanisms

#### 3.1.1. Size Exclusion: Precise Rejection Based on Uniform and Tunable Pore Sizes

**Figure 5:**
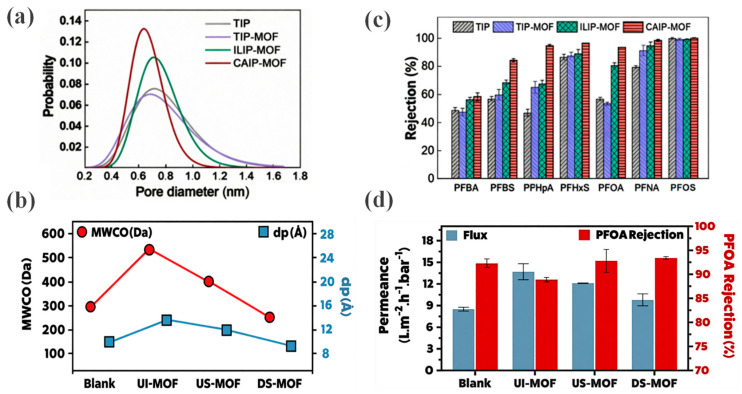
(**a**) Pore size distribution of CAIP-MOF Membrane. Reprinted with copyright permission from Ref. [40] Copyright 2022 American Chemical Society; (**b**) MWCO and average pore diameter (*d*_p_) of UI-MOF/US-MOF/DS-MOF membrane. Reproduced from [39] with Open Access; (**c**) Rejection of representative PFASs. Reprinted with copyright permission from Ref. [40] Copyright 2022 American Chemical Society; (**d**) Water flux and PFOA rejection of the blank and modified membranes. Reproduced from [39] with Open Access.

In the separation of PFAS from water, the size exclusion mechanism is particularly critical for retaining long-chain PFAS molecules with larger molecular sizes, such as PFOA and PFOS. Generally, a lower molecular weight cut-off (MWCO) of the membrane leads to higher rejection rates, as steric hindrance causes the exclusion of specific PFAS with very large molecular sizes [46]. Among OFMs, the CAIP-MOF membrane exhibits the narrowest and left-shifted pore size distribution, with its average pore size precisely controlled to 0.640 nm, significantly smaller than that of conventional TIP membranes (0.754 nm) and other TFN membranes [40] (Figure 5a). The pore sizes of UI-MOF and DS-MOF membranes are 14 ± 0.1 Å and 10 ± 0.1 Å, respectively (Figure 5b). These dimensions are comparable to or smaller than the Stokes diameters of most PFAS molecules, enabling highly efficient rejection through an enhanced size exclusion effect [39].

Simultaneously, the amine-derived positive charges introduced by the UiO-66-NCIM nanoparticles endow the CAIP-MOF membrane with a weakly negative surface charge, somewhat weakening the Donnan exclusion effect. Despite this reduction in Donnan exclusion, the CAIP-MOF membrane demonstrates significantly higher rejection rates for various PFAS (from short-chain PFBA to long-chain PFOS) compared to other membranes (Figure 5c), with near-complete (≈100%) rejection of PFOA and PFOS. These results strongly indicate that size exclusion, rather than the Donnan effect, is the primary mechanism responsible for the high PFAS rejection. Similarly, the DS-MOF membrane, with the smallest pore size, achieves the highest PFOA rejection [39] (Figure 5d), highlighting its significant potential for excluding small solute molecules based on size.

From a geometric probability perspective, the likelihood of a solute molecule entering a membrane pore is closely related to the relative scale between its size and the membrane pore size. Smaller and more uniform membrane pores drastically reduce the effective cross-sectional area available for passage when PFAS molecular dimensions approach or exceed the pore size, leading to a significant decrease in permeation probability and enhanced physical exclusion, thereby achieving precise molecular sieving.

Furthermore, beyond the membrane’s pore architecture, the amphiphilic character of PFAS indirectly amplifies size-based rejection by altering their aqueous behavior. The hydrophobic perfluorinated tail drives micellization, enlarging the effective solute diameter so that the species become susceptible to size-exclusion by the membrane [35]. Concurrently, the hydrophilic head group fosters the formation of molecular clusters, further increasing the apparent hydrodynamic radius and elevating the probability of retention by the membrane’s sieving network, thereby intensifying the overall size-exclusion effect [37].

#### 3.1.2. Electrostatic Repulsion: Leveraging Surface Charge and the Donnan Effect

**Figure 6:**
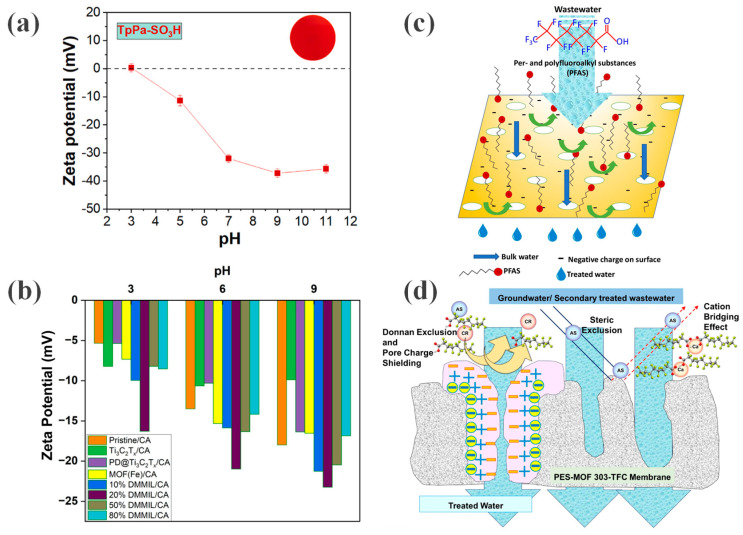
(**a**) zeta potential of the TpPa-SO_3_H membrane. Reprinted with copyright permission from Ref. [43] Copyright 2024 Elsevier B.V.; (**b**) Zeta potential of pristine CA membrane and MMMs. Reproduced from [35] with Open Access; (**c**) PFOA removal mechanism of the DMMIL/CA MMM. Reproduced from [35] with Open Access; (**d**) PFOA/PFOS removal mechanism of the PES-MOF 303-TFC membrane. Reprinted with copyright permission from Ref. [42] Copyright 2025 Elsevier B.V.

When the solute size is smaller than the membrane pore size, electrostatic repulsion (i.e., the Donnan effect) becomes the dominant separation mechanism. Charged membranes repel solutes carrying like charges via electrostatic interactions, preventing their passage through the membrane pores. Under typical aqueous pH conditions, most PFAS compounds (e.g., PFOA and PFOS) exist in anionic forms due to the dissociation of their carboxylic or sulfonic acid head groups. Charged functional groups on the membrane surface can enhance the electrostatic repulsion between the membrane and negatively charged solutes, such as PFAS anions, thereby contributing to PFAS removal [47]. Consequently, constructing membranes with strongly negatively charged surfaces is a key strategy for improving PFAS rejection. For instance, in the case of OFMs, the TpPa-SO_3_H COF membrane prepared by Nguyen et al. [43] features sulfonic acid groups densely grafted onto the COFs. These groups are highly dissociated in water, endowing the membrane surface with a strong negative charge. As indicated in Figure 6a, the zeta potential becomes more negative with increasing pH. Although its physical pore size (1.01–1.46 nm) is larger than some PFAS molecules, the powerful electrostatic repulsion serves as the primary driving force for separation. Minhas et al. [35] fabricated DMMIL/CA membranes with varying DMMIL proportions (10–80%), which were negatively charged across a wide pH range (Figure 6b). The incorporation of highly electronegative nanomaterials further enhanced the membrane’s electronegativity, thereby strengthening the electrostatic repulsion against the similarly negative-charged PFOA molecules (Figure 6c). Similarly, Koli et al. [42] introduced MOF-303 into the PES support layer to increase the negative charge density on the TFC membrane surface, thereby enhancing the rejection of anionic pollutants. As can be seen from Figure 6d, besides size exclusion, electrostatic interactions also contribute to PFAS separation by the TFC membrane.

**Figure 7:**
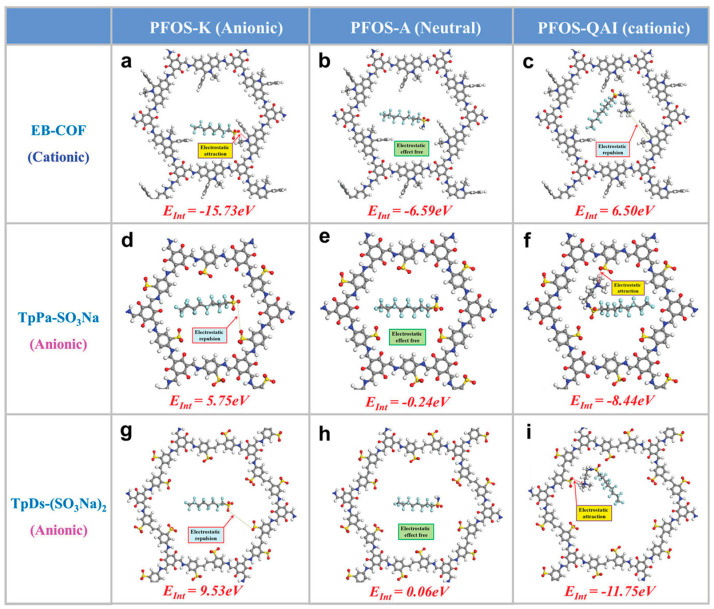
Structural models and *E*_Int_ of the complexes composed of iCOFs and PFASs molecules. Reprinted with copyright permission from Ref. [36] Copyright 2022 Wiley-VCH GmbH: (**a**–**c**) *E*_Int_ of EB-COF with anionic PFOS-K, neutral PFOS-A, and cationic PFOS-QAI; (**d**–**f**) *E*_Int_ between TpPa-SO_3_Na and different PFASs molecules; (**g**–**i**) *E*_Int_ between TpDs-(SO_3_Na)_2_ and different PFASs molecules.

Furthermore, to gain deeper insight into the intrinsic mechanism governing the selective separation of differently charged molecules by composite membranes, Zhang et al. [36] fabricated a bilayer composite membrane (PAN@iCOFs) using two COFs with distinct charge characteristics and confirmed the predominant role of electrostatic interactions. They employed Density Functional Theory (DFT) to calculate the interaction energy (*E*_Int_) of different molecules with the membrane material surface (Figure 7), theoretically quantifying the strength of the electrostatic interactions and thereby elucidating the underlying mechanism of the selective separation.

#### 3.1.3. Adsorption and Specific Host–Guest Interactions

Beyond physical sieving and macroscopic electrostatic effects, the rich chemical environments within the pores of MOFs, COFs, and HOFs provide a variety of specific host-guest interactions, enabling the capture and retention of PFAS molecules through adsorption [48,49,50].

**Figure 8:**
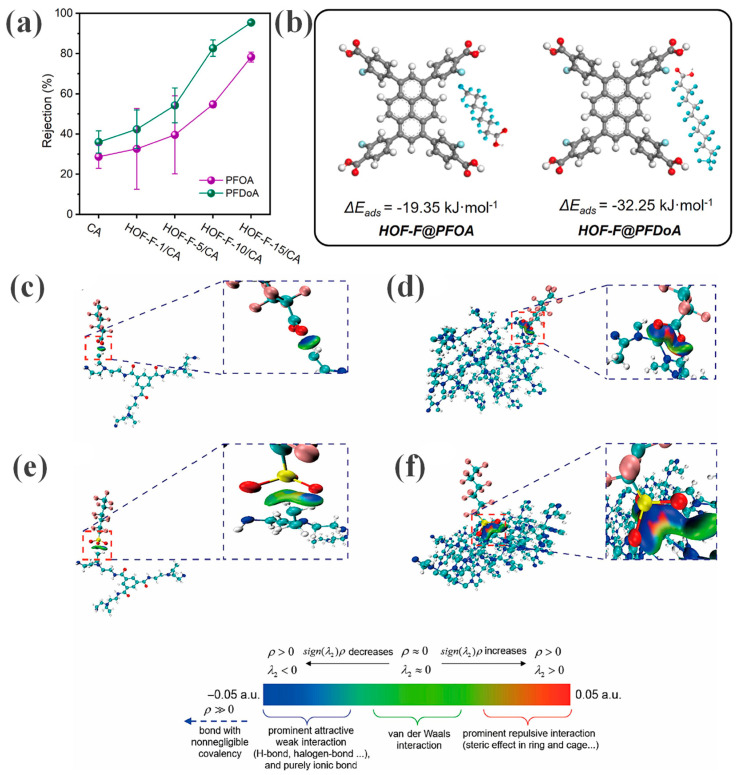
(**a**) PFAS rejection performance of the HOF–F/CA MMMs. Reprinted with copyright permission from Ref. [37] Copyright 2025 Elsevier B.V.; (**b**) HOF–F/PFAS adsorption energy. Reprinted with copyright permission from Ref. [37] Copyright 2025 Elsevier B.V. Interaction isosurfaces for (**c**) PFHxA–PA, (**d**) PFHxA–ZIF-L, (**e**) PFHxS–PA, and (**f**) PFHxS–ZIF-L. Reprinted with copyright permission from Ref. [38] Copyright 2024 Elsevier B.V.

In the HOF-F/CA membrane prepared by Lin et al. [37], strong F–F interactions between the fluorinated (F–C) functional groups of HOF–F and the fluorocarbon chains of PFOA and perfluorododecanoic acid (PFDoA) serve as the driving force, leading to their preferential adsorption. The exposed carboxyl groups in HOF–F further enhance the adsorption efficiency by forming hydrogen bonds with the functional groups of PFAS molecules. DFT calculations confirmed the favorability of these interactions, yielding adsorption energies of −19.35 and −32.25 kJ·mol^−1^ for PFOA and PFDoA on HOF–F, respectively (Figure 8a,b). In the ZIF-L/PEI membrane fabricated by Bi et al. [38], the -CF_2_ and -SO_3_ groups of PFAS molecules act as hydrogen bond acceptors, forming hydrogen bonds with the hydrogen atoms (hydrogen bond donors) of the N-H groups in the amide linkages (-CO-NH-) of the polyamide layer. Upon introduction of PFAS into the solution, hydrogen bonds are established between the -CF_2_/-SO_3_ groups and the PA layer. Figure 8c,d illustrate the hydrogen bonding interactions between the PA membrane and perfluorohexanesulfonic acid (PFHxS) or perfluorohexanoic acid (PFHxA), respectively. In Figure 8e,f, the isosurfaces between ZIF-L and PFASs are depicted in dark blue, demonstrating that the incorporation of ZIF-L significantly enhances the hydrogen bonding interactions between the membrane and the PFASs. These findings collectively indicate that the enhanced hydrogen bonding network is a key factor in increasing the filtration resistance of PFAS.

In the process of PFAS separation by OFMs, mechanisms such as size exclusion, electrostatic repulsion, and adsorption/host-guest interactions often operate synergistically, collectively determining the ultimate removal efficiency of PFAS. Among them, the MOF-303-modified TFC membrane prepared by Koli et al. [42] utilized both electrostatic repulsion and size exclusion effects by constructing a negatively charged and dense separation layer. Studies by Zhang et al. [41] demonstrated that the removal of short-chain PFAS by their PA-modified MOF membrane benefits from multiple mechanisms: the relatively small pore size ensured size exclusion, the high surface negative charge density enhanced electrostatic repulsion, while the MOF (Cu-TCPP) provided adsorption capacity through interactions such as hydrogen bonding, hydrophobic interactions, and π–π interactions between its surface functional groups and PFAS molecules. Here, adsorption compensates for the limitations of relying solely on physical sieving for the retention of short-chain PFAS.

### 3.2. Catalytic Destruction: Beyond Separation

This mechanism differs from the separation membranes described above, as this strategy primarily utilizes advanced oxidation processes. It involves using the membrane as a catalyst; by integrating catalytically active sites into the membrane structure, pollutants can be simultaneously separated and degraded in situ into harmless or less toxic substances. Furthermore, the catalytic reactions of these catalysts can continuously degrade pollutants adsorbed or attached to the membrane surface, thereby mitigating membrane fouling [51].

**Figure 9:**
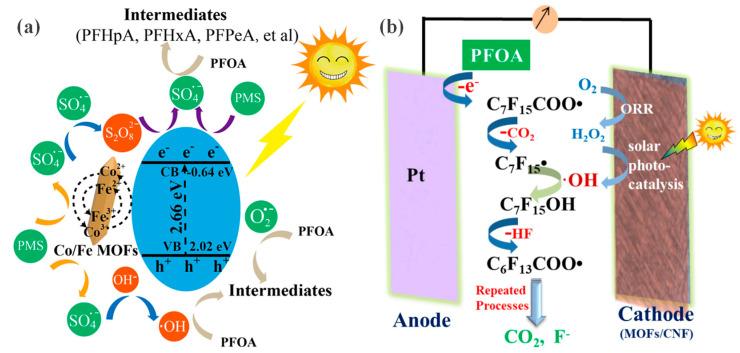
(**a**) Reaction mechanism of the PMS/Membrane/Solar Light process. Reprinted with copyright permission from Ref. [53] Copyright 2021 Elsevier B.V.; (**b**) PFOA mineralization mechanism by the MOFs/CNF SPEF system. Reprinted with copyright permission from Ref. [52] Copyright 2021 Elsevier B.V.

Hou et al. [52] developed a composite membrane based on bimetallic MOF (Co/Fe) and carbon nanofibers (CNF) for the degradation of PFOA. As shown in Figure 9a, this composite membrane introduces catalytically active metals at the metal nodes of the MOF. These sites can generate highly reactive sulfate radicals (SO_4_^−^) and hydroxyl radicals (·OH) in the presence of light and peroxymonosulfate (PMS). These radicals can attack and break the exceptionally stable C–F bonds in PFOA molecules, ultimately mineralizing them into CO_2_, F^−^, and water. In another study, Wang et al. [53] constructed a more complex solar photo-electro-Fenton (SPEF) system, whose core is a MOFs/CNF composite membrane. This system utilizes a bifunctional catalytic cathode, where the CNF membrane not only acts as a flexible substrate supporting the MOF but also synthesizes H_2_O_2_ in situ via the two-electron oxygen reduction reaction (ORR). Subsequently, the photocatalytic activity of the MOFs rapidly converts H_2_O_2_ into ·OH (Figure 9b). Under light and in the presence of oxidants, this material system can efficiently degrade PFOA, providing a clear proof of concept for developing such catalytic membranes. Both studies analyzed the degradation intermediates using ultra-high performance liquid chromatography—mass spectrometry (UHPLC-MS), revealing that PFOA degradation proceeds via stepwise decarboxylation, forming shorter-chain perfluorocarboxylic acids.

The exceptional performance of OFMs in PFAS removal stems from the synergistic interplay of multiple separation mechanisms enabled by their unique structures. These mechanisms incorporate additional chemical and physiochemical interactions, allowing for more precise and efficient separation—and even catalytic degradation—of PFAS molecules. Based on this understanding, the rational design of material properties such as pore size, surface charge, and chemical functionality at the molecular scale paves the way for developing highly efficient separation membranes tailored to different PFAS contamination scenarios.

## 4. PFAS Removal Performance of OFMs

### 4.1. High-Performance Membranes: Combining High Rejection with Ideal Permeability

Some reported membranes could achieve a favorable balance between PFAS rejection and water permeability. For example, the ZIF-L/PEI (M-5) MOF membrane exhibits rejection rates of 98.5% and 95.9% for PFOS and PFOA, respectively, while maintaining a water flux of 47.6 LMH/bar. Its separation performance arises from the synergistic contributions of size exclusion, electrostatic repulsion, and hydrogen bonding. However, this membrane shows an optimal filler loading (5 wt%); excessive loading (10 wt%) leads to aggregation of ZIF-L nanoparticles and the formation of interfacial defects, which increases water flux to 74.7 LMH/bar but significantly compromises PFAS rejection. Moreover, its performance is sensitive to operating pressure. At 5 bar, the rejection of PFHxA and PFHxS decreases to 47.2% and 62.1%, respectively, highlighting its limitations under high-pressure conditions [38]. The TpPa-SO_3_H COF membrane exhibits a PFOS rejection exceeding 99%, while maintaining rejection rates of 90–95% for PFOA, PFBS, and PFBA, with a water flux of 19.9–37.5 LMH/bar. Its high performance is primarily attributed to the strong electrostatic repulsion (Donnan effect) generated by the densely distributed sulfonic acid groups on the surface, despite its intrinsic pore size (1.01–1.46 nm) being larger than the molecular dimensions of PFAS [43].

### 4.2. Highly Selective/Specialized Separation Membranes: Targeting Specific Application Scenarios

Many high-performance membranes do not rely on a single mechanism but instead achieve efficient PFAS removal through the synergy of multiple mechanisms. For example, the Cu-TCPP MOF–PAm membrane demonstrates high removal efficiencies for 11 PFAS, including five short-chain and six long-chain species (e.g., PFBA 84.2%, PFOA 92.5%, PFOS 93.8%). Its performance arises from the combined contributions of size exclusion, electrostatic repulsion, and multiple adsorption interactions between the MOF and PFAS molecules, including hydrogen bonding, hydrophobic interactions, and π–π stacking. This multi-mechanism synergy—particularly the adsorption interactions—effectively enhances the capture of short-chain PFAS with smaller molecular sizes [41].

From Table 1, it can be seen that the PAN@iCOFs COF membrane exhibits the highest water flux among all membrane materials. Its water flux (749.4–838.5 LMH/bar) is higher than that of the pristine PAN substrate membrane (715.4 LMH/bar), indicating that incorporating iCOFs significantly enhances membrane permeability. This improvement may stem from the good hydrophilicity of iCOFs and the additional mass transfer pathways formed within the polymer matrix. The positively charged (EB-COF) and negatively charged (TpPa-SO_3_Na/TpDs-(SO_3_Na)_2_) iCOFs work synergistically within the membrane. Through strong electrostatic interactions (Donnan effect), they efficiently attract and capture PFAS molecules carrying opposite charges. As a result, the removal efficiencies for charged PFAS—whether anionic PFOS-K or cationic PFOS-QAI—exceed 99.9%. However, because the pore sizes of the iCOFs (1.4–2.38 nm) are larger than the size of PFAS molecules, the membrane shows almost no retention capability for neutral PFAS (e.g., PFOS-A) [36].

The separation performance of the HOF-F/CA membrane toward PFAS arises from the synergistic action of multiple mechanisms, among which F–F interactions play a pivotal role. DFT calculations show that the membrane exhibits a significantly higher adsorption energy for long-chain PFDoA (−32.25 kJ·mol^−1^) compared to short-chain PFOA (−19.35 kJ·mol^−1^). This stronger interaction directly translates into higher rejection, with PFDoA achieving a rejection rate of 95.4%, whereas PFOA reaches 78.3% [37].

The Co-Fe MOF catalytic membranes adopt a fundamentally different “destruction” strategy rather than mere separation. Through catalytic degradation (PMS activation/photo-electro-Fenton), they can achieve PFOA degradation rates of 89.6% to 99% within hours, fundamentally avoiding the challenge of concentrate handling. This makes them suitable for in situ treatment of high-concentration PFAS pollution sources [52,53].

### 4.3. Membranes with Performance Trade-Offs: Areas for Future Development

The DMMIL/CA membrane can remove not only PFAS but also other organic and inorganic pollutants in real wastewater; however, its maximum pure water flux is only 7.8 LMH/bar, and the flux is even lower for contaminated water. In synthetic wastewater, the optimal membrane achieves a PFOA removal rate of 91.4%, which drops to approximately 60% in real wastewater. This decline is likely due to the presence of other charged species (e.g., anions and cations) in real wastewater, which alter the solution’s charge environment and may weaken or interfere with the membrane’s electrostatic repulsion toward PFAS. Membrane performance is highly sensitive to the loading of DMMIL nanofillers: a 20% DMMIL/CA membrane exhibits reduced rejection due to excessive porosity, whereas an 80% DMMIL/CA membrane suffers from low flux due to insufficient porosity. These observations indicate that precise process control is critical during membrane fabrication [35]. The Ag-MOF series membranes clearly illustrate the “rejection-permeability” trade-off. Employing different modification strategies increased the PFOA rejection from 88.9% (UI-MOF) to 93.4% (DS-MOF), but the water permeance correspondingly decreased from 13.7 LMH/bar to 9.8 LMH/bar [39].

The AlFu MOF/PVA modified PTFE membrane case illustrates the critical impact of membrane material affinity. The pristine hydrophobic PTFE membrane exhibits a 0% PFOA rejection rate due to strong affinity leading to adsorption and diffusion. Incorporating AlFu MOF into a hydrophilic PVA layer effectively inhibits this diffusion, increasing the PFOA rejection to 65.6%. However, this value remains substantially lower than its rejection rates for PFOS and PFHxS (>99.7%), underscoring the significant performance variation across different PFAS species [34].

The CAIP-MOF membrane, governed primarily by a precise size exclusion mechanism with an average pore size of 0.640 nm, achieves near-complete rejection (>99%) of long-chain PFOS and PFOA but shows significantly lower rejection for smaller short-chain PFAS, such as only 50% for PFBA and 60% for PFBS [40]. This highlights the limitation of relying solely on size exclusion for removing short-chain PFAS.

Compared with purely separation-based membranes, catalytic membranes offer a significant advantage: they can achieve permanent degradation of PFAS rather than merely concentrating them. For instance, the Co-Fe MOF/CNF system can achieve 99% PFOA degradation within 120 min through complete mineralization [52]. However, catalytic membranes also face challenges, including catalyst instability (e.g., metal leaching), energy input requirements (e.g., light or electricity), and the potential formation of intermediate byproducts [51]. Therefore, the choice between separation membranes and catalytic membranes should be closely aligned with the specific application scenario: separation membranes are better suited for treating large volumes of low-concentration PFAS-contaminated water, whereas catalytic membranes are more appropriate for point-source treatment, where the primary goal is the thorough destruction of high-concentration PFAS.

## 5. Challenges and Future Perspectives

The aforementioned studies indicate that through rational structural design and surface modification, OFMs have demonstrated significant potential at the laboratory scale for simultaneously enhancing antifouling capabilities and long-term stability. For instance, MOF-303-TFC membranes and the Ag-MOF series membranes maintain high flux recovery rates (>94%) after multiple fouling–cleaning cycles [39,42], while the Cu-TCPP MOF-PAm membrane exhibits no performance deterioration during continuous operation for up to 120 h [41]. These findings lay a solid foundation for the application of OFMs in complex water treatment environments. However, translating these membranes from laboratory research to large-scale practical applications still faces a series of formidable challenges, and future studies should prioritize the following directions.

### 5.1. Materials Targeting High Durability and Strategies for Enhanced Stability

Future research should focus on the design and synthesis of organic frameworks with intrinsically higher chemical and hydrothermal stability. As systematically outlined in the design of stable MOFs, the core strategy lies in combining thermodynamic stability with kinetic reinforcement. Structural integrity is ensured by constructing strong metal–ligand coordination bonds, introducing framework interpenetration, and increasing connectivity [54]. Simultaneously, continued exploration of novel strategies for membrane structure enhancement is warranted, including polymer crosslinking, incorporation of inorganic nanomaterials, and the construction of multilayer composite architectures, with the aim of improving mechanical strength and resistance to compaction.

### 5.2. Rapid and Scalable Fabrication Routes

Developing innovative membrane fabrication processes is essential for industrial deployment. Promising directions include exploring rapid synthesis methods—such as microwave-assisted or photo-initiated reactions—to reduce the preparation time of framework materials or membranes; advancing continuous, roll-to-roll membrane fabrication techniques, including spraying, slot-die coating, and printing, to replace traditional batch laboratory methods [55]; and optimizing key steps such as interfacial polymerization to achieve fast and precise control over membrane structure and performance.

In addition, rational design approaches, exemplified by computational modeling and machine learning, offer the potential to significantly accelerate the development of high-performance OFMs. Recent studies have introduced a “data–mechanism” dual-driven design framework, in which machine learning is used to resolve interactions between pollutants and membranes at the functional-group level, thereby guiding targeted material design [56]. Such approaches can predict the stability of new frameworks and their interactions with PFAS, enabling the identification of optimal candidates prior to synthesis and substantially shortening the research and development cycle.

### 5.3. Sustainability and Environmental Compatibility

As OFMs technologies continue to advance, their environmental friendliness has become an increasingly important concern. Future research should explore greener synthesis routes, such as producing OFMs in aqueous media or under mild conditions to minimize the use of hazardous solvents.

### 5.4. Pilot-Scale Long-Term Studies Targeting Practical Applications

To date, most studies remain at the laboratory scale, employing synthetic wastewater for short-term testing. There is an urgent need for more long-term (spanning several months or even years) pilot-scale investigations using real water matrices, such as industrial effluents, landfill leachate, or contaminated groundwater. Such studies can comprehensively evaluate the actual separation performance, antifouling capability, flux recovery after cleaning, and long-term operational stability of membranes under complex water quality conditions, including the effects of competing ions and natural organic matter [57]. This represents an indispensable step for validating the practical applicability of the technology and obtaining essential parameters for engineering design.

### 5.5. Regulation-Driven Materials Design: Emphasis on Short-Chain PFAS

Globally, regulations on PFAS are tightening rapidly, with an increasingly clear trend of expanding control from conventional long-chain PFAS (e.g., PFOA and PFOS) to short-chain species (e.g., PFBA and PFBS) [58]. This regulatory shift places explicit and urgent demands on future OFMs design: it is essential to develop broad-spectrum membrane materials capable of efficiently removing both short-chain and long-chain PFAS.

## Figures and Tables

**Table 1:** Performance Comparison of Representative OFMs for PFAS Removal.

Membrane	Organic Framework	PFAS Type	PFAS Rejection/Degradation Rate (%)	Water Permeance (LMH/bar)	Primary Separation Mechanisms	Reference
MOF-303-TFC	MOF	PFOS, PFOA ^a^	92.1 ± 0.6, 91.8 ± 0.6	7.91–14.72	Size Exclusion, Electrostatics	[42]
Cu-TCPP MOF-PAm	MOF	11 types of PFAS	84.2–94.0	21.4	Size Exclusion, Adsorption, Electrostatics	[41]
AlFu MOF/PVA (PSA5)	MOF	28 types of PFAS ^b^	>98.4 (Total)	41	Electrostatics, Diffusion Hindrance	[34]
ZIF-L/PEI (M-5)	MOF	6 types of PFAS	35.5–100	47.56	Size Exclusion, Electrostatics, Hydrogen Bonding	[38]
Ag-MOF (UI-MOF)	MOF	PFOA	88.9	13.7	Size Exclusion, Electrostatics	[39]
Ag-MOF (US-MOF)	MOF	PFOA	92.8	12.1	Size Exclusion, Electrostatics	[39]
Ag-MOF (DS-MOF)	MOF	PFOA	93.4	9.8	Size Exclusion, Electrostatics	[39]
DMMIL/CA	MOF	PFOA, PFHpA, PFHxA ^c^	55–91.4	7.8	Hydrogen Bonding, Electrostatics	[35]
CAIP-MOF (UiO-66-NCIM)	MOF	8 types of PFAS	50–99	18.7	Size Exclusion	[40]
Co-Fe MOF(PVA)	MOF	PFOA	89.6 (Deg. 3 h)	-	Catalytic Degradation	[52]
Co-Fe MOF (CNF)	MOF	PFOA	99.0 (Deg. 2 h)	-	Catalytic Degradation	[53]
TpPa-SO_3_H COF	COF	PFOS, PFOA, PFBS, PFBA	90–99	11.5–37.9	Electrostatics, Size Exclusion	[43]
PAN@iCOFs	COF	PFOS-K/PFOS-QAI, PFOS-A	>99, <3	749.4–838.5	Dual-Charge Electrostatics	[36]
HOF-F/CA	HOF	PFDoA, PFOA	95.4, 78.3	175	F-F Interaction, Size Exclusion	[37]

^a^ Tested by groundwater and secondary wastewater; data represents mean ± standard deviation. ^b^ Tested by landfill leachate with 28 PFAS compounds. ^c^ Tested by synthetic wastewater and real wastewater.

## Data Availability

The original contributions presented in this study are included in the article. Further inquiries can be directed to the corresponding author.
